# The antibacterial effect of *Ziziphora clinopodioides *essential oil and nisinagainst *Salmonella typhimurium *and *Staphylococcus aureus* in doogh, a yoghurt-based Iranian drink

**Published:** 2016-09-15

**Authors:** Yasser Shahbazi

**Affiliations:** *Department of Food Hygiene and Quality Control, Faculty of Veterinary Medicine, Razi University, Kermanshah, Iran.*

**Keywords:** Doogh, Nisin, *Salmonella typhimurium*, *Staphylococcus aureus*, *Ziziphora clinopodioides*

## Abstract

Doogh is the most popular and commonly consumed yoghurt-based Iranian drink. The aim of this study was to investigate the antibacterial effects of *Ziziphora clinopodioides* essential oil (ZEO) at 0.10 and 0.20% concentrations, nisin at 250 and 500 IU mL^-1^, and their combination against *Salmonella typhimurium *and *Staphylococcus* *aureus* in doogh during storage at 4 ˚C for  9 days. Nine batches were studied as follows: control: no ZEO or nisin added, A: 0.10% ZEO, B: 0.20% ZEO, C: 250 IU mL^-1^ nisin, D: 500 IU mL^-1 ^nisin, E: 0.10% ZEO + 250 IU mL^-1 ^nisin, F: 0.10% ZEO + 500 IU mL^-1 ^nisin, G: 0.20% ZEO + 250 IU mL^-1 ^nisin and H: 0.20% ZEO + 500 IU mL^-1 ^nisin. Based on gas-chromatography and mass spectrometry, carvacrol (65.22%), thymol (19.51%), *p*-cymene (4.86%) and *ɣ*-terpinene (4.63%) were the major components of ZEO. The populations of *S. typhimurium* and *S.* *aureus* in samples treated with all concentrations of the ZEO and nisin were kept below 1 log CFU mL^-1 ^on day 5 of storage, while the count of *S*. *typhimurium* and *S.* *aureus* was found as 2.72 ± 0.02 and 2.21 ± 0.00 log CFU mL^-1^ on day 5 for untreated samples, respectively. The ZEO separately and in combination with nisin, was very effective against these two common food-borne pathogens. The ZEO alone and in combination with nisin could be considered as a potential strong antimicrobial agent that can be used for the growth inhibition of aforementioned bacteria in food products especially doogh.

## Introduction

Doogh is the most commonly consumed Iranian yoghurt drink with a long history of manufacturing. Traditionally, it is prepared by full fat yoghurt, water, salt and sweet-smelling herbs mixing in special leather bag known as Mashk in Persian language. Salt at a maximum level of 1 g per 100 g and sweet-smelling herbs such as zizifore, mint, oregano and thyme are added to impart flavor. In recent years with increasing demand for doogh consumption in different parts of Iran, it is commercially produced from homogenized and pasteurized skim milk (90.00 to 95.00 ˚C for 10 min) and various commercial essential oils (EOs).^1^^-^^3^

*Salmonella *species are one of the most important food-borne pathogenic bacteria, causing diversity of diseases including typhoid fever, gastroenteritis and septicemia. In recent years, an increase in notifications of gastroenteritis outbreaks due to *S. typhimurium* has been reported throughout the world.^4^^,^^5^ Milk and dairy products, juices, fruits, vegetables, meat and fish products are widely recognized as important sources of *Salmonella *spp*. *contamination and vehicles of Salmonellosis.^5^ Moreover, *Staphylococcus aureus* can survive and grow in various foods. It is a potential public health hazard because entero-toxigenic strains of coagulase-positive *S. aureus *can produce Staphylococcal enterotoxins leading to food poisoning.^6^^,^^7^ Numerous reported Salmonellosis and Staphylococcal food poising outbreaks were associated with contaminated milk and dairy products.^8^

Antimicrobial effects of the EOs and extracts of medicinal plants such as oregano, clove, cinnamon, garlic, rosemary, mint and basil, or their components were reported against food-borne pathogens in various food models.^9^^-^^11^ *Ziziphora clinopodioides *(Known as Kakouti Kohi in Persian) is an edible medicinal plant belonging to *Laminaceae* family widely grows in Iran and Turkey.^12^^-^^14^ In Iran, this plant is extensively used as a spice in a wide variety of foods especially milk and dairy products such as yoghurt, cheese and doogh.^15^

The antimicrobial efficacy of EOs against food-borne pathogens may be influenced by several important factors including their chemical composition, method of EO extraction, different bacterial species and model of food.^16^ The EOs combination with other antimicrobial agents such as nisin, may help their concentration reduction. Nisin is produced by *Lactococcus lactis* subsp. *Lactis *or *Streptococcus uberis* and has inhibitory effect against Gram-positive bacteria by permeating the cytoplasmic membrane.^17^ Also, it has been reported recently that nisin can act against Gram-negative bacteria by a synergistic effect with other antimicrobial factors such as EOs.^4^^,^^17^^-^^21^ It has been applied as a food preservative additive since the 1940s and currently approved as a food additive in over 50 countries.^22^

In comparison to many other EOs of medicinal plants, few data are available about the antibacterial influence of *Z. clinopodioides *EO alone or in combination with nisin in food model systems. However, several *in vitro* studies have demonstrated that *Z. clinopodioides *EO possesses potential activity as an antibacterial agent.^13^^-^^15^ Microbial growth inhibition of *Z. clinopodioides *EO has been evaluated by disc diffusion and broth micro-dilution methods and it was found that EO has high antibacterial effects against *Bacillus subtilis*, *Bacillus cereus *and *Listeria monocytogenes.*^13^ It has been shown that EO of *Z. clinopodioides *collected from Khorasan Razavi province, Iran, exhibits strong anti-bacterial activities against *Staphylococcus epidermidis, S. aureus, Escherichia coli *and *B. subtilis*.^15^ Moreover, to the best of the author’s knowledge, there is no study involving doogh manufactured in Iran illustrating the presence and/or survival of important pathogenic bacteria such as *S. aureus* and *Salmonella* spp. Hence, the aim of the present study was to investigate the effects of *Z. clinopodioides* EO alone and in combination with nisin on survival of *S. typhimurium* and *S. aureus *inoculated in doogh samples during storage under refrigerated temperature (4.00 ± 1.00 ˚C) for nine days. 

## Materials and Methods

**Plant material. **The fresh leaves of *Z. clinopodioides* plant were harvested from Gilan-e-Gharb, Kermanshah province, western Iran in March to July 2014. The plant was identified and authenticated in Faculty of Agriculture, Razi University, Kermanshah, Iran. Voucher specimen (No. 6816) of the plant was deposited in the herbarium of the Research Center of Natural Resources of Tehran, Iran. The fresh leaves were extensively washed with distilled water (20.00 ˚C) and dried for two weeks at room temperature (25.00 ± 2.00 ˚C). Then, the dried leaves were used for subsequent extraction. 

**Isolation of EO.** *Z. clinopodioides* EO was obtained by hydro-distillation in a clevenger-type apparatus during 3 hr.^23^ Fine powdered tissue (100 g) were used for isolation of the EO. Basically, the extraction procedure consisted in streaming the vapor generated in a boiler through the bed where the plants were put on. Then, the solute was dragged and after that, it was condensed by contact with a cold fluid. The EO was recovered by phase separation, collected on top of the distillate, dried over anhydrous sodium sulfate (Na_2_SO_4_; Merck, Darmstadt, Germany) and kept in darkness in a sealed vial preserved at 4.00 ± 1.00 ˚C until GC-MS analysis and further use. 

**Gas chromatography–mass spectrometry (GC–MS) analysis of EO. **The analysis of *Z. clinopodioides *EO was done using a GC (Thermo Quest Corp., Austin, UK) coupled with mass spectrometer detector (GC-MS; Thermo Quest, Finningan, UK) and equipped with DB5 capillary column (30.00 m length × 0.25 mm inner diameter, 0.25 µm film thickness) and HP-5MS (5.00% phenyl methyl silicone and 95.00% dimethylpolysiloxane).^23^ For GC-MS analysis, electron ionization energy of 70 eV was applied over a scan range of 40-500 amu. Helium was a carrier gas with a constant flow rate of 1.20 mL per min. The initial temperature was 50.00 ˚C and kept for 3 min, it gradually increased up to 265 ˚C at increment of 2.50 ˚C per min, and finally held at 265 ˚C for 6 min. Analysis of the EO was also conducted by gas chromatograph (Thermo Quest). The capillary column and temperature condition were similar with gas chromatograph coupled with a mass spectrometer described above. 

**Identification of chemical compounds. **The most volatile chemical compounds of the EO were identified by comparison between their retention indices, retention indices of published data, standard mass spectral fragmentation pattern (Wiley/NBS Pak v.7, 2003) and the National Institute of Standards and Technology (NIST; v.2.0, 2005). The gas chromatography peak area normalization of the three injections was expressed as mean percentage of individual EO composition. 

**Preparation of nisin. **Nisin with a label activity of 10^4 ^IU g^-1^ was purchased from Sigma (Dorset, UK). A stock solution of nisin was prepared by dissolving of nisin in 0.02 M HCl. Then, it was centrifuged at 1500 *g* for 20 min, filtered by 0.22 µm pore filter (Sigma) and stored at – 20 ˚C until use.^24^ Prior to test, the stock solution was thawed at 25 ˚C and diluted in sterile water to a concentration of 250 and 500 IU mL^-1 ^nisin. 

**Test microorganisms. **The strains used in this study were *S. typhimurium* (ATCC 14028) and *S. aureus *(ATCC 6538). Lyophilized cultures of the microorganisms were obtained from the culture collection of the Iranian Research Organization for Science and Technology (IROST), Tehran, Iran. Before the test, the bacterial strains were sub-cultured twice in Brain Heart Infusion broth (BHI; Merck) medium and incubated at 37 ˚C for 18 hr. The density of bacterial cultures needed for inoculation of doogh samples was examined using spectrophotometer at 600 nm. The determination of inoculum dose (10^5 ^CFU mL^-1^) was also assessed using triplicate plate count on BHI agar medium.^3^

**Preparation of doogh. **Full-fat yoghurt was purchased from a local store of Kermanshah, west of Iran. Before the test, the total solid, pH, total lipid, total sugar, protein and ash were measured. Then, doogh sample was prepared by addition of yoghurt (3.50 g 100 g^-1^ total lipid, 3.52 g 100 g^-1^ protein, 0.80 g 100 g^-1^ ash, 14.30 g 100^ -1^ total solid and 5.32 g 100 g^-1^ total sugar) and water at the ratio of 1:1, followed by thorough mixing for 30 sec. After this step, NaCl was added to doogh sample at the ratio of 1 g 100 g^-1^ and sample was gently stomached for 30 sec at room temperature.^2^

In order to evaluate the antibacterial activities of the EO and nisin, a sufficient amount of doogh was prepared and tested using two-fold minimum inhibitory concentration (MIC) values for EO and nisin. The MIC values were determined using broth micro-dilution method.^10^ Briefly, 5.00% (v/v) dimethyl sulfoxide (DMSO; Merck) was used as an emulsifier and 0.05% (w/v) agar-agar (Merck) was used as a stabilizer of the EO. Then, different concentrations of the EO (0.0125, 0.025, 0.05, 0.10, 0.25, 0.50, 1.00, 1.50 and 2.00%) and nisin (3.75, 7.50, 15.00, 30.00, 60.00, 125, 250, 500 and 1000 IU mL^-1^) were set up using 96-well sterile micro-dilution plates with U-bottom wells. Then, 180 µL of BHI broth containing different concentrations of EO or nisin and 20 µL of the final bacterial inoculums (1 × 10^5^ CFU mL^-1^) were added to each well. As a positive control, the same amount of BHI broth containing bacterial inoculums without EO or nisin was added to well. Moreover, in each experiment, negative controls and BHI broth containing DMSO and EO or nisin were considered. Then, the contents of plates were shaken at 250 rpm for 30 sec and incubated at 37.0 ˚C for 24 hr. The MIC was described as the lowest concentration of the EO or nisin that prevents the growth of the micro-organisms. The experiment was carried out in triplicate. For both microorganisms, the MICs of EO and nisin were 0.05% and 125 IU mL^-1^, respectively. As described above, two-fold MIC values were considered for evaluating of antimicrobial activities of the EO and nisin in doogh samples. 

Nine batches (Batch A-Batch H) were studied as follows: control (no EO or nisin added), A (0.10% *Z. clinopodioides* EO), B (0.20% *Z. clinopodioides* EO), C (250 IU nisin), D (500 IU nisin), E (0.10% *Z. clinopodioides* EO + 250 IU nisin), F (0.10% *Z. clinopodioides* EO + 500 IU nisin), G (0.20% *Z. clinopodioides* EO + 250 IU nisin) and H (0.20% *Z. clinopodioides* EO + 500 IU nisin). Then, doogh samples were inoculated with *S. typhimurium *and *S. aureus* cultures at the level of 10^5 ^CFU mL^-1^. After homogenization for 30 sec, all samples were kept at refrigerated temperature (4.00 ± 1.00 ˚C) until measure-ments were made. The microbial analysis of doogh samples was conducted at day 0, 1, 3, 5, 7 and 9. The experiment was repeated in triplicate. 

**Microbiological analysis. **The batches were aseptically opened, 10 mL of each samples were diluted with 0.10 g 100 mL^-1 ^sterile buffered peptone water (Merck) and surface plated onto *Salmonella Shigella* agar (SS agar; Merck) and Baird Parker agar (Merck) media separately.^5,6^ Plates were incubated at 37.00 ± 2.00 ˚C for 24 hr. Results were expressed as log CFU mL^-1^.

**Sensory evaluation. **The sensory effects of adding *Z. clinopodioides* EO and nisin to doogh samples were evaluated using an acceptance test. A panel of seven judges experienced in dairy product evaluation was used for sensory analysis. Panelists were asked to evaluate odor and flavor of samples. Acceptability of samples was estimated using an acceptability scale ranging from 10 to 1 with 10 corresponding to the most liked sample and 1 corresponding to the least liked sample.^25^

**Statistical analysis. **Data were analysed using SPSS (Version 16.0, SPSS Inc., Chicago, USA). Mean values and standard deviation of each experiment were calculated and subjected to analysis of variance. Tukey's test at 95.00% confidence interval was used to determine mean differences among the treatments. 

## Results

**Chemical composition of** ***Z. clinopodioides*** **EO. **The GC-MS analysis resulted in identification of 24 compounds accounting for 99.65% of the whole EO (Table 1). 

**Table 1:** Essential oil composition of *Ziziphora clinopodioides *identified by gas chromatography mass spectrometry

Compound name	Composition (%)	RT^a^ (min)	KI^b^
α-Thujene	0.26	11.33	927
α-Pinene	0.27	11.71	934
Camphene	0.13	12.61	952
β-Pinene	0.06	14.06	981
1-Octen-3-ol	0.08	14.32	986
Myrcene	0.51	14.62	992
α-Phellandrene	0.13	15.58	1010
α-Terpinene	0.79	16.11	1021
p-Cymene*	4.86	16.62	1030
Limonene	0.10	16.77	1033
β-Phellandrene	0.11	16.89	1036
ɣ-Terpinene*	4.63	18.31	1063
cis-Sabinene hydrate	0.07	19.02	1077
Terpinolene	0.08	19.69	1089
Linalool	0.13	20.50	1105
Borneol	0.61	24.36	1183
Terpinene-4-ol	0.48	24.70	1190
α-Terpineol	0.08	25.49	1206
Carvacrol methyl ether	0.04	27.38	1246
Thymol*	19.51	29.61	1293
Carvacrol*	65.22	30.57	1315
E-Caryophyllene	1.07	35.47	1427
Spathulenol	0.12	42.10	1590
Caryophyllene oxide	0.31	42.30	1595
Other	0.08	-	-
Total	99.65	-	-

a Retention time;

b Kovats index.

* The dominant compounds are indicated in bold.

The major components were phenolic compounds including carvacrol (65.22%), thymol (19.51%), *p-*cymene (4.86%) and ɣ-terpinene (4.63%).

**Survival of** ***S. typhimurium*** **and** ***S. aureus*** **in doogh during storage at 4** **˚C. **Survival of *S. typhimurium* and* S. aureus *in doogh following nine different treatments is shown in Tables 2 and 3 as a function of storage time and antibacterial effect of *Z.* *clinopodioides *alone and in combination with nisin. The initially recorded populations of 5.00 log CFU mL^-1 ^of *S. typhimurium* and *S. aureus *reached to 2.72 ± 0.02 and 2.21 ± 0.00 log CFU mL^-1 ^by the day nine in control doogh samples, respectively. With regard to *S. typhimurium*, samples treated with nisin at 250 and 500 IU mL^-1 ^alone and in combination with 0.10% and 0.20% EO presented populations of the pathogen significantly different than those of control samples through-out storage at 4.00 ˚C (*p *< 0.05), indicating significant antimicrobial activity of nisin and EO against the pathogen in doogh samples. Based on our results, there was no significant difference between samples treated with the combination of EO at 0.10% or 0.20% and nisin at 250 IU mL^-1 ^and those of samples treated with the combination of EO at 0.10% and 0.20% and nisin at 500 IU mL^-1 ^(*p *> 0.05). The populations of *S. typhimurium* in samples treated with all concentrations of EO and nisin were kept below 1 log CFU mL^-1 ^on day 5 of storage, while the count was found as 2.72 ± 0.02 log CFU mL^-1 ^on day 5 for untreated samples. Regarding *S. aureus, *samples treated with the combination of EO at 0.20% and nisin at 500 IU mL^-1^, showed populations of *S. aureus* significantly lower than those of samples treated with other groups (*p *< 0.05). Also, our samples treated with EO and nisin, separately and in combination, showed populations of the pathogen significantly lower than untreated samples (*p *< 0.05).

**Sensory properties. **Acceptability scores (odor and flavor properties) of doogh samples for all different treatments are shown in Table 4. There were significant differences in the odor and flavor of treated samples as compared with the untreated control (*p *< 0.05). It should be noted that *Z. clinopodioides *(at 0.10% concentration) odor and flavor were very excellent and did not hamper sensory evaluation of samples. Likewise, nisin at 250 and 500 IU mL^-1 ^did not affect sample sensory properties.

**Table 2.** Effect of *Ziziphora clinopodioides *essential oil, nisin and their combination on *Salmonella typhimurium *in doogh stored at 4 ˚C.

**Day**	**Control**	**Essential oil (%)**		**Nisin (IU mL** ^-1^ **)**		**Essential oil** ** (%) + Nisin (IU mL** ^-1^ **)**			
		**0.10**	**0.20**	**250**	**500**	**0.10 + 250**	**0.10 + 500**	**0.20 + 250**	**0.20 + 500**
**0**	5.00 ± 0.00^aA^	5.00 ± 0.00^aA^	5.00 ± 0.00^aA^	5.00 ± 0.00^aA^	5.00 ± 0.00^aA^	5.00 ± 0.00^aA^	5.00 ± 0.00^aA^	5.00 ± 0.00^aA^	5.00 ± 0.00^aA^
**1**	4.70 ± 0.28^aA^	4.42 ± 0.00^bB^	4.23 ± 0.02^bcB^	5.15 ± 0.07^adA^	4.88 ± 0.14^dA^	4.01 ± 0.00^cB^	3.98 ± 0.00^cB^	3.98 ± 0.00^cB^	3.76 ± 0.01^cB^
**3**	4.39 ± 0.07^aB^	3.89 ± 0.00^bC^	3.50 ± 0.07^eC^	4.06 ± 0.08^bB^	3.79 ± 0.13^beB^	3.29 ± 0.05^ceC^	3.19 ± 0.14^cdeC^	3.10 ± 0.00^cC^	2.78 ± 0.00^dC^
**5**	2.72 ± 0.02^aC^	ND	ND	2.10 ± 0.00^bC^	ND	ND	ND	ND	ND
**7**	ND	ND	ND	ND	ND	ND	ND	ND	ND
**9**	ND	ND	ND	ND	ND	ND	ND	ND	ND

Lowercase superscript letters in the same row indicate significant differences (*p *< 0.05).

Uppercase superscript letters in the same column indicate significant differences (*p *< 0.05).

ND: Not detected.

**Table 3:** Effect of *Ziziphora clinopodioides *essential oil, nisin and their combination on *Staphylococcus aureus* in doogh stored at 4 ˚C.

**Day**	**Control**	**Essential oil (%)**		**Nisin (IU mL** ^-1^ **)**		**Essential oil** ** (%) + Nisin (IU mL** ^-1^ **)**			
		**0.10**	**0.20**	**250**	**500**	**0.10 + 250**	**0.10 + 500**	**0.20 + 250**	**0.20 + 500**
**0**	5.00 ± 0.00^aA^	5.00 ± 0.00^aA^	5.00 ± 0.00^aA^	5.00 ± 0.00^aA^	5.00 ± 0.00^aA^	5.00 ± 0.00^aA^	5.00 ± 0.00^aA^	5.00 ± 0.00^aA^	5.00 ± 0.00^aA^
**1**	4.95 ± 0.04^aA^	3.90 ± 0.01^bB^	3.06 ± 0.00^dB^	4.80 ± 0.00^gB^	4.49 ± 0.00^hB^	3.84 ± 0.01^bB^	3.73 ± 0.02^cB^	3.25 ± 0.00^eB^	2.96 ± 0.01^fB^
**3**	3.70 ± 0.00^aB^	3.23 ± 0.02^bdC^	2.82 ± 0.02^eC^	3.22 ± 0.07^bC^	2.91 ± 0.1^cdeC^	2.96 ± 0.00^cC^	2.79 ± 0.06^deC^	2.30 ± 0.00^fC^	ND
**5**	2.21 ± 0.00^aC^	ND	ND	ND	ND	ND	ND	ND	ND
**7**	ND	ND	ND	ND	ND	ND	ND	ND	ND
**9**	ND	ND	ND	ND	ND	ND	ND	ND	ND

Lowercase superscript letters in the same row indicate significant differences (*p *< 0.05).

Uppercase superscript letters in the same column indicate significant differences (*p *< 0.05).

ND: Not detected.

**Table 4:** Acceptability scores (odor and flavor) of doogh samples in different treatments

**Day**	**Control**	**Essential oil (%)**		**Nisin (IU mL** ^-1^ **)**		**Essential oil** ** (%) + Nisin (IU mL** ^-1^ **)**			
		**0.10**	**0.20**	**250**	**500**	**0.10 + 250**	**0.10 + 500**	**0.20 + 250**	**0.20 + 500**
**0**	10.0 ± 0.00^a^	10.0 ± 0.00^a^	9.50 ± 0.00^a^	10.0 ± 0.00^a^	10.0 ± 0.00^a^	10.0 ± 0.00^a^	10.0 ± 0.00^a^	9.43 ± 0.02^a^	9.51 ± 0.09^a^
**1**	10.0 ± 0.00^a^	10.0 ± 0.00^a^	9.50 ± 0.00^a^	10.0 ± 0.00^a^	10.0 ± 0.00^a^	10.0 ± 0.00^a^	10.0 ± 0.00^a^	9.50 ± 0.02^a^	9.55 ± 0.00^a^
**3**	7.00 ± 0.72^b^	9.02 ± 0.21^b^	8.61 ± 0.20^b^	9.01 ± 0.00^b^	9.00 ± 0.06^b^	9.06 ± 0.15^b^	9.23 ± 0.20^b^	8.62 ± 0.16^b^	8.52 ± 0.08^b^
**5**	5.12 ± 0.18^c^	8.17 ± 0.34^c^	6.92 ± 0.30^c^	8.10 ± 0.12^c^	8.10 ± 0.23^c^	8.01 ± 0.28^c^	8.10 ± 0.10^c^	6.91 ± 0.11^c^	7.01 ± 0.23^c^
**7**	4.56 ± 0.10^c^	7.11 ± 0.00^d^	6.71 ± 0.10^c^	7.33 ± 0.11^d^	7.12 ± 0.18^d^	7.11 ± 0.32^d^	7.21 ± 0.36^d^	6.52 ± 0.22^c^	6.52 ± 0.16^d^
**9**	3.33 ± 0.16^d^	6.91 ± 0.00^e^	5.10 ± 0.10^d^	6.91 ± 0.01^d^	6.92 ± 0.09^d^	6.76 ± 0.11^d^	6.82 ± 0.35^d^	5.12 ± 0.21^d^	5.22± 0.03^e^

## Discussion

The major components of the EO were phenolic compounds including carvacrol (65.22%), thymol (19.51%), *p-*cymene (4.86%) and ɣ-terpinene (4.63%). Chemical composition of plants EOs and spices can vary depending on different methods used for EO extraction, geographical conditions, climate and seasonal variations and stage of the plant growth.^6^^,^^12^^,^^27^^,^^28^ In this regard, it has been reported that the most abundant compounds of *Z. clinopodioides* EO collected from the Erzurum-Palandoken mountain of Turkey are phenolic compounds including pulegone (31.86%), 1,8-cineole (12.21%), limonene (10.48%), menthol (9.13%), β–pinene (6.88%), menthone (6.73%), piperitenone (5.30%) and piperitone (4.18%).^13^ Also, it was found that pulegone (44.50%), terpineol (14.50%), methyl acetate (10.90%), iso-neomenthol (7.10%) and 1,8-cineole (4.10%) are the main components of the EO obtained from Mashhad, Khorasan Razavi province (North East of Iran).^15^ The greater content of phenolic compounds was also reported previously. Based on these findings, carvacrol (8.70%) and thymol (53.60%) were the major compounds of the EO of *Z. clinopodioides* harvested from Lorestan province of Iran confirming the results of present study.^12^ It is believed that higher amounts of phenolic compounds exhibit higher antimicrobial effects against micro-organisms. The most important reason of the strong antibacterial activities of carvacrol and thymol is the acidic nature of their hydroxyl group and involvement in the formation of hydrogen bonds.^29^

The antibacterial effects of *Z. clinopodioides *EO and nisin, separately and in combination, against* S. typhimurium *and *S. aureus* in doogh have not been reported yet. Previous studies reported that combination of EOs and nisin has a greater effect than EO or nisin separately against food-borne pathogens such as *S. typhimurium*,* S. aureus*, *Salmonella enteritidis* and *E. coli* O157:H7.^30^^,^^31^

In the current research, it was found that *Z. clinopodioides *EO, along with various combinations of nisin, has an enormous influence in reducing the growth of *S. typhimurium* and* S. aureus*. The basic compounds of *Z. clinopodioides* EO such as carvacrol, thymol, ɣ-terpinene and *p*-cymene (Table 1) were reported to provide antimicrobial properties against Gram-positive and Gram-negative bacteria.^12^^,^^13^^,^^15^ Hence, in addition to antimicrobial effect of nisin, this reduction may be caused by constituents present in the *Z. clinopodioides* EO. The mechanism of combination effects of nisin and various EOs is not fully understood. It seems that EO enhances the effect of nisin by increasing the number of pores in the phospholipid bilayer membrane structure and also the size of the pores formed. However, several researchers reported that the combined use of nisin and various EOs may be affected by some factors such as pH, NaCl concentration and incubation temperature.^32^

According to results of this work, following doogh samples storage increase, a significant reduction in the count of *S. typhimurium* and *S. aureus* was observed. In addition to antibacterial effects of nisin and EO, several factors may contribute to reduction of these pathogens during storage, such as presence of lactic acid bacteria. The progressive production of some compounds such as bacteriocin, hydrogen peroxide and volatile compounds by lactic acid bacteria during storage of dairy products is well documented. A number of studies have shown the inhibitory effects of these compounds against food-borne pathogens.^33^^,^^34^ On the other hand, the pH could be a key factor in decrease of survival and growth of *S. typhimurium and S. aureus *in dairy products such as doogh.^35^

Acceptability scores (odor and flavor properties) of doogh samples in all different treatments are shown in Table 4. There were significant differences in the odor and flavor of treated samples as compared with untreated control (*p *< 0.05). It should be noted that *Z. clinopodioides *(at 0.10% concentration) odor and flavor were very excellent and did not hamper sensory evaluation of samples. Likewise, nisin at 250 and 500 IU mL^-1^ did not affect sample sensory properties. Our results are in agreement with previous studies.^25^^,^^26^

In conclusion, in the present research, *Z. clinopodioides* EO gathered from Kermanshah province, west of Iran contains high amount of carvacrol, thymol, *p*-cymene and ɣ-terpinene. Based on our findings, the EO separately and in combination with nisin was very effective against two common food-borne pathogens, *S. typhimurium* and *S. aureus*. Hence, this EO alone and in combination with nisin could be considered as a potential strong antimicrobial agent that can be used for the growth inhibition of various bacteria in food products such as doogh. 
